# Photoisomerization of Linear and Stacked Isomers of a Charged Styryl Dye: A Tandem Ion Mobility Study

**DOI:** 10.1021/jasms.1c00264

**Published:** 2021-11-17

**Authors:** Eduardo Carrascosa, James N. Bull, Emilio Martínez-Núñez, Michael S. Scholz, Jack T. Buntine, Evan J. Bieske

**Affiliations:** † School of Chemistry, The University of Melbourne, Parkville, Victoria 3010, Australia; ‡ School of Chemistry, Norwich Research Park, University of East Anglia, Norwich NR4 7TJ, United Kingdom; § Departamento de Química Física, Universidade de Santiago de Compostela, 15782 Santiago de Compostela, Spain

**Keywords:** ion mobility mass spectrometry, action spectroscopy, isomerization, dye, chemical master equation

## Abstract

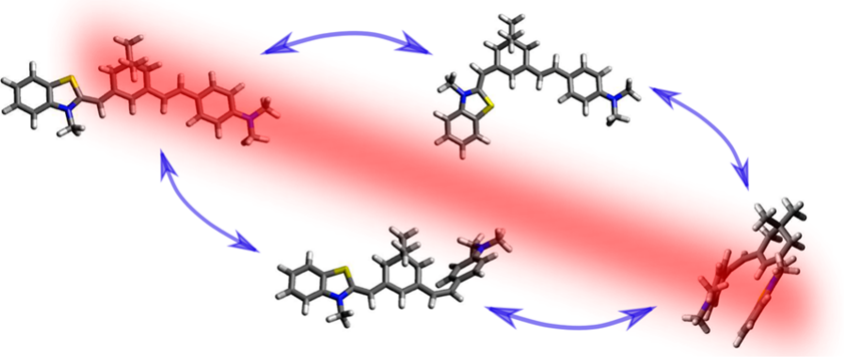

The photoisomerization behavior of styryl 9M, a common dye used in material sciences, is investigated using tandem ion mobility spectrometry (IMS) coupled with laser spectroscopy. Styryl 9M has two alkene linkages, potentially allowing for four geometric isomers. IMS measurements demonstrate that at least three geometric isomers are generated using electrospray ionization with the most abundant forms assigned to a combination of *EE* (major) and *ZE* (minor) geometric isomers, which are difficult to distinguish using IMS as they have similar collision cross sections. Two additional but minor isomers are generated by collisional excitation of the electrosprayed styryl 9M ions and are assigned to the *EZ* and *ZZ* geometric isomers, with the latter predicted to have a π-stacked configuration. The isomer assignments are supported through calculations of equilibrium structures, collision cross sections, and statistical isomerization rates. Photoexcitation of selected isomers using an IMS-photo-IMS strategy shows that each geometric isomer photoisomerizes following absorption of near-infrared and visible light, with the *EE* isomer possessing a S_1_ ← S_0_ electronic transition with a band maximum near 680 nm and shorter wavelength S_2_ ← S_0_ electronic transition with a band maximum near 430 nm. The study demonstrates the utility of the IMS-photo-IMS strategy for providing fundamental gas-phase photochemical information on molecular systems with multiple isomerizable bonds.

## Introduction

Molecules containing photoisomerizable subunits are commonly used as molecular triggers or sensors in technological and biomedical applications.^1^ A desire to develop better photoswitches has motivated numerous experimental and theoretical studies focused on their photophysical behavior. Desirable characteristics for molecular photoswitches include high photoisomerization quantum yield, controllable reversibility, fatigue resistance, large structural change upon photoisomerization, high solubility, and facile chemical functionalization. Recently, researchers have devoted substantial efforts toward developing thermodynamically stable and water-soluble photoswitches that absorb light in the biooptical window,^2^ potentially allowing them to be incorporated in photoswitchable enzymes and proteins, and light-activated drugs for photopharmacological applications.

Common molecular photoswitches based on *E*/*Z* photoisomerizable bonds include azobenzenes and stilbenes,^3−5^ indigoids,^6−8^ hybrid switches containing both azo and alkene linkages,^9^ and Stenhouse adducts.^10,11^ Styryl dyes are another class of photoswitchable molecules, although they are used less frequently in photoswitching applications. Styryl dyes are widely used as sensitizers in solar cells and in the photographic industry,^12^ are common laser dyes,^13,14^ and have been proposed as labeling units for biomedical applications.^15^ Styrylpyridines, the aza-analogues of stilbene, have been thoroughly investigated theoretically^16−18^ and experimentally^19^ due to their potential to propel molecular machines,^20^ drive spin changes in transition organometallic complexes,^21,22^ and act as DNA staining agents.^23^ More complex styryls, including styryl 7 and styryl 9M (S9M), which have two or more isomerizable bonds, have been proposed as efficient fluorescent probes for bioimaging, biosensing, and medical diagnosis applications.^15,24−27^ The attraction of S9M for various biotechnological applications stems partly from its high two-photon absorption cross-section in the near-infrared, enabling its use as a hyperspectral fluorescent probe.^28−30^ The potential applications for styryl dyes have prompted studies of their photoexcitation, fluorescence and photoisomerization dynamics in solution.^31−38^ Many styryl dyes exhibit strong solvatochromism and pH-dependent photoabsorption.^29^

**Figure 1:**
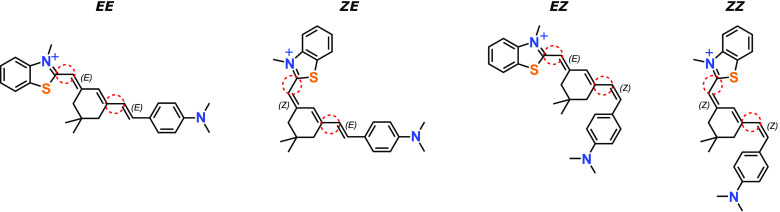
Four geometric isomers of S9M. For each geometric isomer, rotation around the single bonds (circled) results in additional conformers/rotamers (see the Supporting Information for details). Note that the lowest energy structure for the *ZZ* isomer has a stacked geometry (shown more clearly in Figure 2).

To understand the intrinsic characteristics of styryl dyes and the role of solvation in altering their properties, it is desirable to characterize the electronic absorptions and photoisomerization behavior of isolated dye molecules. Here, we use tandem ion mobility spectrometry (IMS) coupled with laser spectroscopy to investigate the charged styryl dye S9M in the gas phase, focusing on its electronic spectrum and its photoisomerization response over the visible spectral range. As shown in Figure 1, S9M is a hexamethine–hemicyanine dye consisting of an electron-donating dimethylaminophenyl group attached to a methylbenzothiazolium electron-withdrawing group through a polymethine chain. There are two alkene linkages, each of which may possess *E* and *Z* configurations, leading to four geometric isomers. By irradiating the target S9M ions between two drift tube IMS stages, we can select a particular target isomer and distinguish photoproduct isomers. This approach has been used previously to investigate photoinduced structural changes for a range of molecular ions in the gas phase.^39−42^ The situation is relatively simple for charged molecules that have only two geometric isomers with significantly different collision cross sections, which can, for example, be interconverted through *E–Z* photoisomerization around a single double bond.^43^ For molecules with several photolabile bonds such as S9M, the situation is more complicated as exposure to light could cause transformations between several different geometric isomers. Perhaps the clearest example of a charged molecule with two photoisomerizable double bonds investigated using this approach is Congo Red, which has two equivalent double bonds, and where *EE*, *EZ*, and *ZZ* isomers were selectively probed using tunable visible light.^40^ It was found that absorption of a single photon provoked reversible isomerization around one double bond and that conversion of *EE* to *ZZ* required consecutive absorption of two photons. Energetic collisions with buffer gas molecules were found to induce conversion of all isomers to the most stable *EE* isomer. The situation for S9M is more complicated than for Congo Red as the two photoisomerizable bonds are not equivalent and there are four rather than three geometric isomers.

## Materials and Methods

### Experimental Procedure

The S9M ions were investigated using a home-built tandem ion mobility spectrometer that has been described previously.^44^ S9M (Sigma-Aldrich, no further purification) was dissolved in methanol at a concentration of 0.1 mM and electrosprayed into an ion funnel (IF1) and injected (100 μs pulses) into a drift region filled with N_2_ buffer gas at a pressure of ∼6 Torr with a drift field of 40 V cm^–1^. The RF drive voltage applied to IF1 could be adjusted to allow sampling of nascent electrosprayed ion isomeric populations (IF1 low) or to collisionally excite the ions (IF1 high), potentially inducing collisional isomerization before the ions entered the drift region. In the drift region, the isomers were separated in space and time according to their collision cross section (CCS) with N_2_ buffer gas. A second ion funnel (IF2) at the end of the drift region radially gathered the ions before they passed through an orifice into an octupole ion guide and quadrupole mass filter where they were mass selected and detected by a Channeltron detector. The detector was connected to a multichannel scaler, which was used to generate a histogram of ion counts against arrival time, corresponding to an arrival time distribution (ATD).

Photoisomerization experiments using the IMS-photo-IMS strategy involved selecting a packet of ions using a Bradbury-Nielsen ion gate situated half way along the drift region^45,46^ and irradiating the mobility-selected ions with tunable wavelength light from an optical parametric oscillator (OPO, EKSPLA NT342B, 20 Hz). Photoisomers were separated from the parent isomer in the second stage of the drift region. The photoisomerization response was quantified by measuring “light-on” and “light-off” ATDs, the difference between which corresponded to the photoaction ATD. Photoisomerization action spectra were derived by plotting the photoisomer yield, normalized to light fluence, as a function of wavelength.

Collision-induced transformations of selected S9M isomers were investigated by passing mobility-selected ions through a short collision zone situated immediately after the photoisomerization zone.^40,46^ The collision region consists of two parallel grids separated by 3 mm across which the potential difference could be adjusted to produce electric fields ranging from 60 to 700 V cm^–1^. High electric fields cause energetic collisions between ions and buffer gas molecules, potentially inducing isomerization on the ground electronic state potential energy surface. Equivalent isomers formed through collisional activation in the collision zone or through photoactivation in photoisomerization zone have essentially the same arrival time at the detector.

Solution absorption spectra of S9M in methanol and chloroform were measured using a Varian Cary 50 Bio spectrophotometer and quartz cuvette (1 cm path length).

### Theoretical Evaluation

The structures and relative energies of S9M isomers were calculated at the ωB97X-D/cc-pVDZ level of density functional theory using the Gaussian16.B01 software package.^47^ Vibrational frequency analysis confirmed that the optimized geometries were true potential energy minima. Ground-state isomerization barriers between conformers/rotamers and stilbene-type cyclization were investigated using transition-state barrier calculations using the Quadratic Synchronous Transit (QST2) path approximation and conformer-search algorithms implemented in the automated reaction discovery program AutoMeKin.^48−50^ Single-point energy calculations on the minimum and transition state geometries were performed at the DLPNO-CCSD(T)/cc-pVTZ level of theory using ORCA 4.2.1.^51^ Vertical excitation energies were calculated at the DF-CC2/aug-cc-pVDZ level of theory using MRCC (Feb 2019 release).^52^

Collision cross sections (CCSs) were calculated for the minimum energy structures using the trajectory method in a version of the MOBCAL package^53^ parametrized for N_2_ buffer gas. Input charge distributions for each structure were computed using the Merz–Singh–Kollman scheme constrained to reproduce the electric dipole moment. The number of computed trajectories was sufficient to give standard deviations of ±1 Å^2^ for the calculated CCSs.

Ground-state isomerization processes were modeled by solving the chemical master equation (CME) using MESMER6.0 (Master Equation Solver for Multi Energy well Reactions) software package.^54^ The rovibrational properties of isomers and transition states, required for the rate coefficient calculations, were determined at the ωB97X-D/cc-pVDZ level of theory. In the CME modeling, energy transfer through collisions with buffer gas was modeled with an exponential down model with an average energy removed per collision of ⟨Δ*E*_down_⟩ = 200 cm^–1^, which has been used in previous studies.^40,55,56^ The bath gas was N_2_ and the collision frequencies were obtained using a Lennard-Jones (LJ) potential with parameters: σ = 3.7047 Å and ε = 84.942 cm^–1^ for N_2_,^57^ and σ = 12.695 Å, and ε = 91.168 cm^–1^ for S9M. The S9M parameters were obtained by fitting N_2_–S9M interaction energies calculated at the semiempirical PM6-D3H4 level of theory assuming LJ combining rules with a LJ potential function. Two types of CME simulations were performed: (1) the S9M ions were given an average thermal energy corresponding to *T* = 300 K, (2) ions were given an average thermal energy corresponding to *T* = 300 K plus the energy imparted through absorption of one or two photons with wavelengths of either 430 or 680 nm.

## Results and Discussion

### Isomers

**Figure 2:**
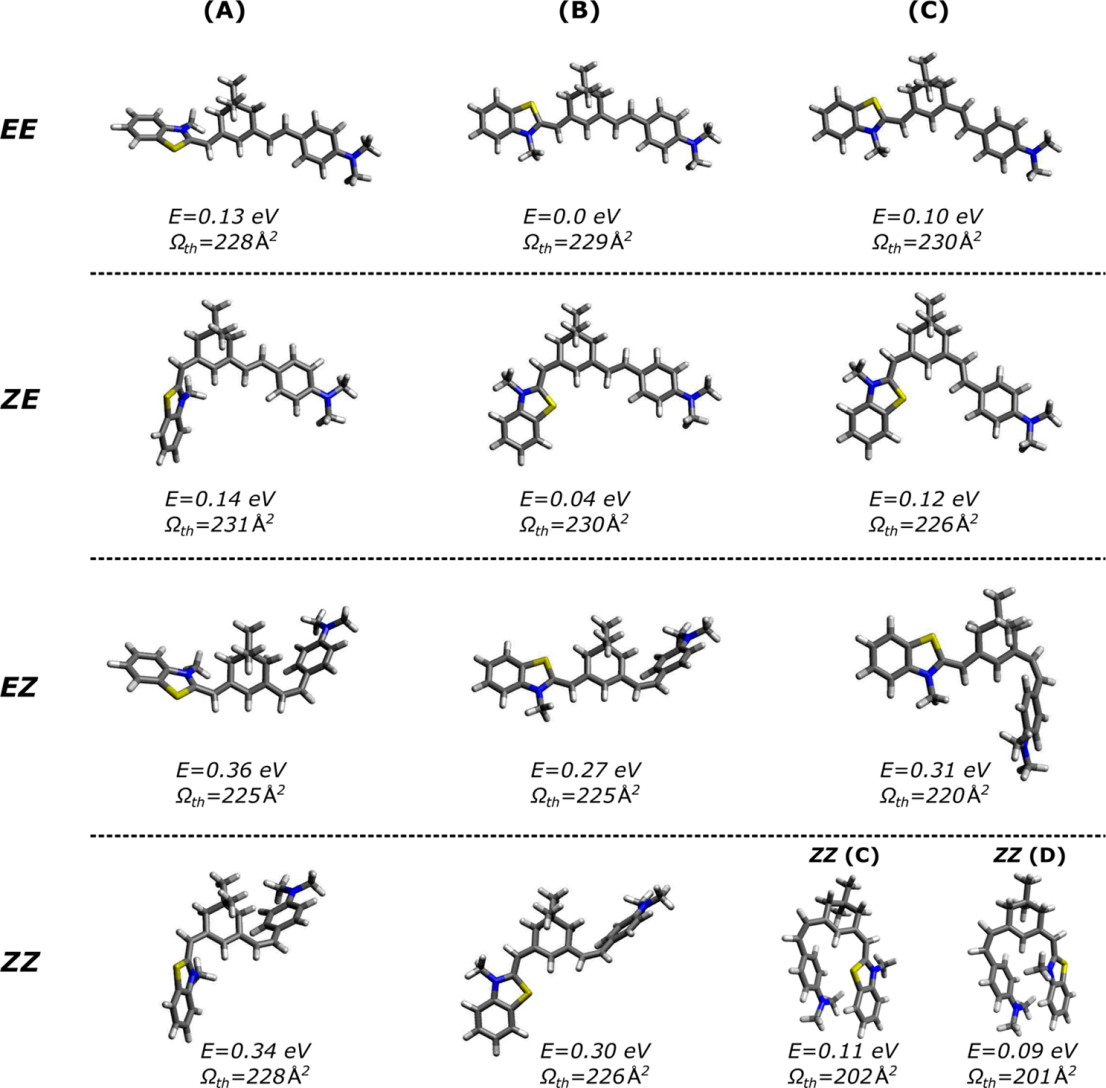
Structures, relative energies [with respect to *EE* (B)], and calculated collision cross sections for low-energy conformers of styryl 9M. The left column (A) corresponds to conformers generated through a single-bond rotation changing the dihedral angle between the methylbenzothiazolium and cyclohexenyl moieties. The right column (C) corresponds to the conformers generated through a single-bond rotation changing the dihedral angle between the cyclohexenyl and (dimethylamino)phenyl moieties. Structures and energies for other conformers are given in the Supporting Information.

**Figure 3:**
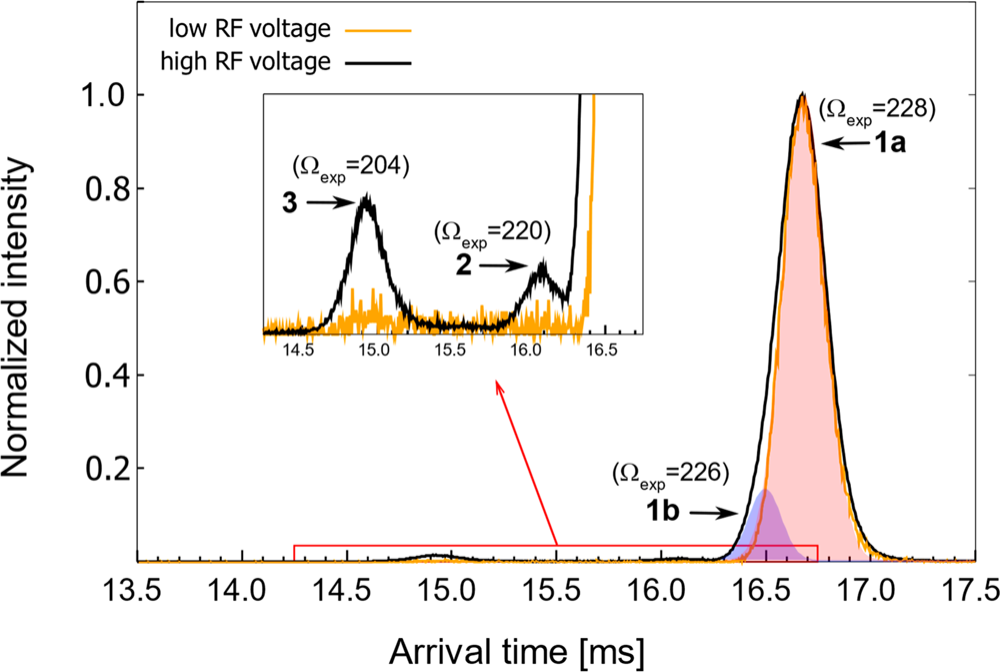
Arrival time distributions (ATDs) for S9M at low (orange) and high (black) RF drive voltages applied to IF1. Experimental collision cross sections (Ω_exp_ with units of Å^2^) are shown for each peak. The shaded areas show the two-component Gaussian fit (**1a** and **1b**) to ATD peak **1** for IF1 high.

Low energy conformations/rotamers for each geometric isomer are shown in Figure 2. The minimum energy conformations for the *EE*, *ZE*, and *EZ* isomers are labeled B. Rotation about the single bond connecting the methylbenzothiazolium moiety and the central cyclohexenyl group (see Figure 1) leads to rotamers A, while rotation about the single bond connecting the cyclohexenyl group with the (dimethylamino)phenyl group (see Figure 1) leads to rotamers C. For the *EE*, *EZ*, and *ZE* isomers, rotamers with rotation about both single bonds (D) lie relatively high in energy; their energies and structures are given in the Supporting Information. Possible three-ring structures resulting from stilbene cyclization were considered^58^ but were ultimately deemed not important in this work based on their much higher relative energies. For the *EE*, *EZ*, and *ZE* isomers, the B rotamers lie lowest in energy. Although *EE*(B) is the lowest energy structure, the *ZE*(B) structure lies only 0.04 eV higher in energy. The lowest energy conformations of the *ZZ* isomer (C and D in Figure 3) have compact, π-stacked geometries with relatively small collision cross sections; similar π-stacked geometries are not possible for the other three geometric isomers. Calculated CCS values (Ω_*th*_) for each structure are included in Figure 3.

### Arrival Time Distributions (ATDs)

ATDs for S9M at low (orange) and high (black) RF drive voltage applied to IF1 are shown in Figure 3. With low RF voltage on IF1, a single ATD peak (peak **1**) was observed with a mobility resolution (*t*/Δ*t* ≈ 70), which is comparable to that for previous ATDs recorded with the instrument.^59^ This suggests that the electrosprayed ions exist in a single isomeric form or as isomers with very similar CCSs. Increasing the RF drive voltage applied to IF1 resulted in a broadening of ATD peak **1** on the short arrival time side and the appearance of two other ATD peaks, peaks **2** and **3**, at shorter arrival time. The broadening of peak **1** suggests collision-induced formation of another isomer with a slightly smaller collision cross-section than the original electrosprayed isomer. To assess the contribution from this isomer, peak **1** in an ATD recorded at high RF voltage was fitted with two Gaussian functions with the parameters for one Gaussian function fixed to values obtained from a fit of peak **1** recorded at low IF1 RF voltage. CCSs for the isomers (peaks **1a**, **1b**, **2**, and **3**) derived using the Mason–Schamp equation and the fitted arrival times are given in Figure 3. Ultimately, we conclude that there are at least four isomers present when the S9M ions are introduced into the drift region with IF1 operating at high RF voltage.

Before assigning the ATD peaks in Figure 3 to particular isomers, we consider interconversion of the S9M isomers and conformers in the IMS drift region through CME modeling, assuming a temperature of *T* = 300 K. The relative abundances of each species after 20 ms (the approximate ion drift time) starting from a pure ensemble of each species is given in Table 1. The modeling predicts that a quasi-equilibrium between the rotamer forms of a given geometric isomer is established after a few milliseconds. The consequence of this relatively rapid interconversion is that several rotamers will contribute to a single ATD peak associated with a given geometric isomer, and that the measured CCS will be an average of the CCSs for the contributing rotamers. For the *EE*, *ZE*, and *EZ* isomers, the main conformers are predicted to be *EE*(B), *ZE*(B), and *EZ*(B), respectively, with minor contributions in each case from the *EE*(C), *ZE*(C), and *EZ*(C) conformers. Due to their higher energies, the A and D conformers are predicted to be present in negligible abundances. For the *ZZ* isomer, the most important conformers are *ZZ*(C) (major) and *ZZ*(D) (minor). The four principal geometric isomers, *EE*, *ZE*, *EZ*, and *ZZ*, are separated by large barriers and according to the CME simulations should not appreciably interconvert during their passage through the drift region. The one exception is that there is slight interconversion between the *EE* and *ZE* isomers.

**Table 1:** CME-Simulated Abundances of S9M Isomers in the Ion Mobility Drift Region.^a^

	product isomer at *T* = 300 K (%)												
initial isomer	*EE*(A)	*EE*(B)	*EE*(C)	*ZE*(A)	*ZE*(B)	*ZE*(C)	*EZ*(A)	*EZ*(B)	*EZ*(C)	*ZZ*(A)	*ZZ*(B)	*ZZ*(C)	*ZZ*(D)
*EE*(A)	–	93	6	–	1	–	–	–	–	–	–	–	–
*EE*(B)	–	93	6	–	1	–	–	–	–	–	–	–	–
*EE*(C)	–	93	6	–	1	–	–	–	–	–	–	–	–
*ZE*(A)	–	1	–	–	93	6	–	–	–	–	–	–	–
*ZE*(B)	–	1	–	–	93	6	–	–	–	–	–	–	–
*ZE*(C)	–	1	–	–	93	6	–	–	–	–	–	–	–
*EZ*(A)	–	–	–	–	–	–	1	77	22	–	–	–	–
*EZ*(B)	–	–	–	–	–	–	1	77	22	–	–	–	–
*EZ*(C)	–	–	–	–	–	–	1	77	22	–	–	–	–
*ZZ*(A)	–	–	–	–	–	–	–	–	–	–	1	78	21
*ZZ*(B)	–	–	–	–	–	–	–	–	–	–	1	78	21
*ZZ*(C)	–	–	–	–	–	–	–	–	–	–	1	78	21
*ZZ*(D)	–	–	–	–	–	–	–	–	–	–	1	78	21

a The CME simulations started with a particular rotamer and isomer at *T* = 300 K and 6 Torr buffer gas pressure (drift region conditions) and were run for 20 ms. A quasi-equilibrium between conformers associated with each isomer was reached in all cases after several milliseconds. Interconversion between *EE*, *ZE*, *EZ*, and *ZZ* isomers is predicted to take much longer than the drift time.

The ATD peaks can be assigned to the S9M isomers shown in Figure 2 by comparing experimental and calculated CCSs (Table 2), and by considering the relative peak intensities together with calculated energies for the isomers. Keeping in mind that each ATD peak corresponds to packets of ions with time-averaged rotamer structures and CCSs, peak **1a** is assigned to the *EE* isomer since this is the most stable form and is predicted to have a large collision cross-section, consistent with the relatively long arrival time. ATD peak **1b** is assigned to the *ZE* isomer, which lies only slightly higher in energy than *EE* and has a calculated CCS within ∼1 Å^2^ of *EE*. The main reason for assigning peak **1a** to *EE* and peak **1b** to *ZE* rather than *vice versa*, is the slightly lower calculated energy of the *EE* isomer, which means that it should be the dominant isomer. The collision cross sections for the two isomers are predicted to be practically the same.

**Table 2:** Calculated Ground-State Energy (*E*_rel_), Calculated Collision Cross-Section (Ω_th_), Experimental Collision Cross-Section (Ω_exp_), and Calculated S_1_ ← S_0_ and S_2_ ← S_0_ Vertical Transition Wavelengths, With Corresponding Oscillator Strengths Indicated in Parentheses

isomer	*E*_rel_ (eV)	Ω_th_ (Å^2^)	ATD peak	Ω_exp_(Å^2^)	S_1_←S_0_ (nm)	S_2_←S_0_ (nm)
*EE*(B)	0.00	229	**1a**	228	669 (2.2)	380 (0.1)
*EE*(C)	0.10	230	**1a**	228	668 (2.2)	393 (0.3)
*ZE*(B)	0.04	230	**1b**	226	667 (1.6)	393 (0.5)
*ZE*(C)	0.12	226	**1b**	226	674 (1.4)	401 (1.0)
*EZ*(B)	0.27	225	**2**	220	660 (1.7)	395 (0.2)
*EZ*(C)	0.31	220	**2**	220	719 (1.0)	407 (1.0)
*ZZ*(C)	0.11	202	**3**	204	700 (0.3)	424 (0.8)
*ZZ*(D)	0.09	201	**3**	204	623 (0.3)	398 (1.1)

Peak **2** is likely to correspond to the *EZ* isomer which has two conformers possessing intermediate collision cross sections (220 and 225 Å^2^, respectively) and relatively high energies, consistent with the observation that peak **2** is weak and only appears when the ions are energized in the first ion funnel. The peak at shortest arrival time (peak **3**) clearly belongs to the *ZZ* isomer, which has two conformers, *ZZ*(C) and *ZZ*(D), with energies 0.11 and 0.09 eV above that of the minimum energy structure, and with calculated cross sections that are much less than those for other isomers. Because of its higher energy, this isomer is only observed when the ions are energized in the first ion funnel. As shown below, the ATD peak assignments are consistent with the photoisomerization action spectra and calculated vertical excitation wavelengths for each isomer.

### Collision-Induced Isomerization

**Figure 4:**
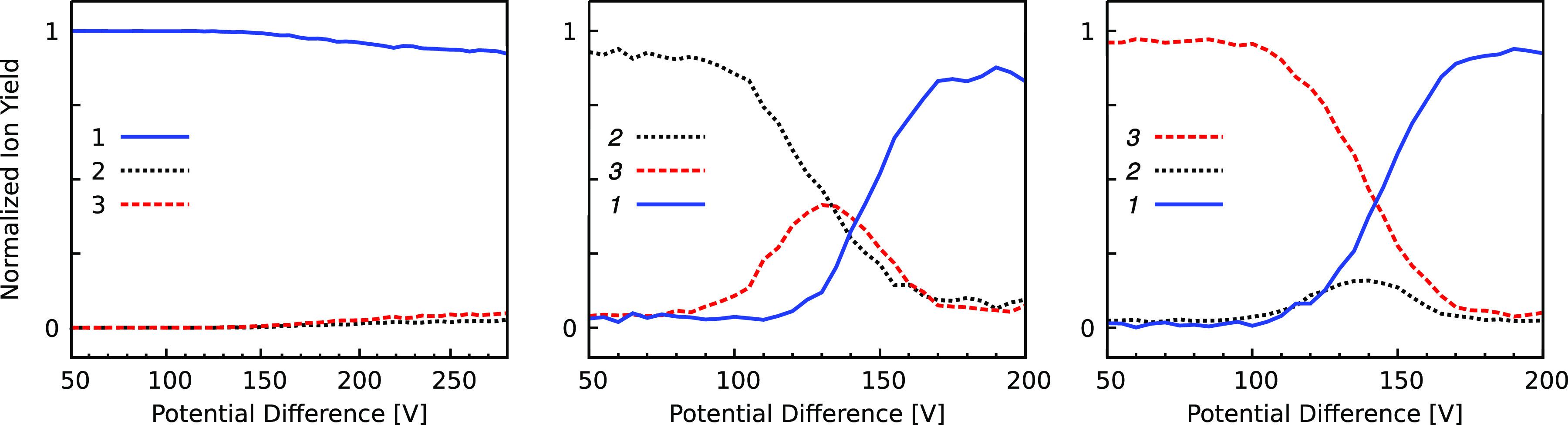
ATD peak areas as a function of potential difference between the grids in the collisional activation zone. An isomer ion packet corresponding to ATD peak **1**, **2**, or **3** (left to right) was selected using the Bradbury Nielsen ion gate immediately before the collision zone. Further details are given in the text.

Collision-induced transformation between S9M isomers was investigated using the IMS–collision–IMS strategy.^40,46^ Collision-induced isomerization branching ratios plotted as a function of the potential difference across the collision zone for ions associated with ATD peaks **1**–**3** are shown in Figure 4. Note that the limited resolution of each IMS stage makes it impossible to individually select the isomers of peaks **1a** and **1b** or to resolve them as collision products. For peak **1** isomers (*EE* and *ZE*), there is a minor conversion to peak **2** and peak **3** isomers (*EE* and *ZE*, respectively) with increasing voltage difference across the collision zone. For ATD peak **2** isomers (*EZ*), there is collision-induced conversion to peak **3** isomers starting at 90 V with maximum yield at 130 V. Peak **1** isomers begin to be formed at ∼130 V and dominate for potential differences exceeding 140 V. For peak **3** isomers (*ZZ*), peak **2** isomers (*EZ*) are generated with collision zone potential differences in the 100–160 V range, with generation of peak **1** isomers (*EE* and *ZE*) dominating for potential differences exceeding 140 V.

**Figure 5:**
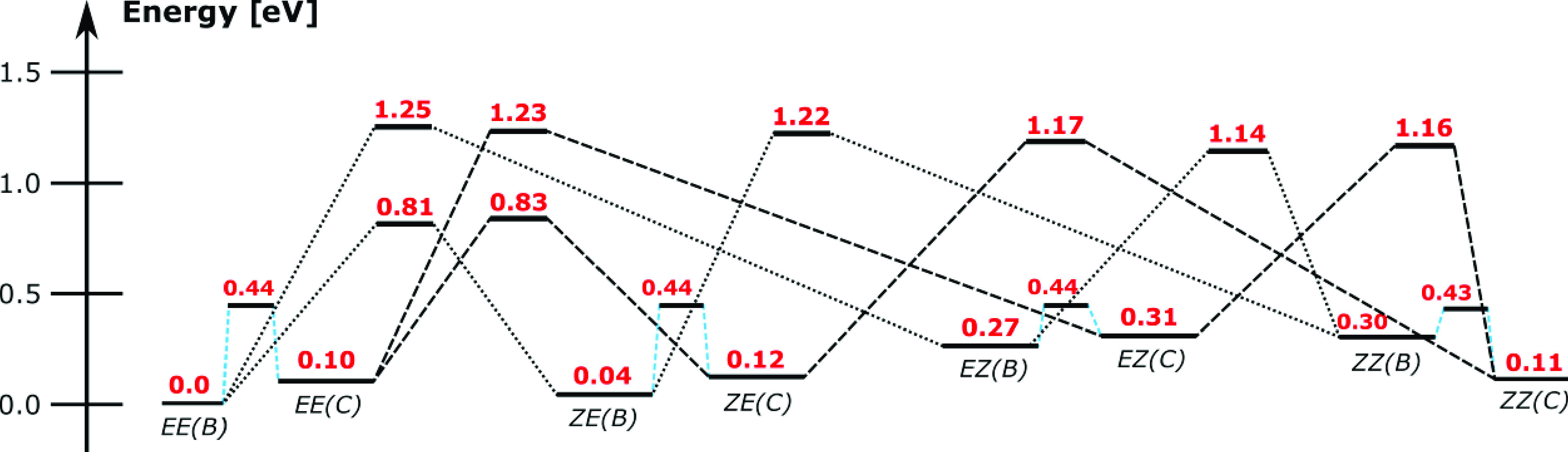
Potential energy diagram linking the more stable structures of S9M (see Figure 2). Energies were computed at the DLPNO-CCSD(T)/cc-pVTZ//ωB97X-D/cc-pVDZ level of theory.

To rationalize these observations, we computed transition-state barriers for interconversion between the (B) and (C) isomers shown in Figure 2 and constructed the potential energy surface shown in Figure 5. The important features are that the *EZ* → *ZZ* barriers are lower than the *EZ* → *EE* barriers, consistent with the observation that *EZ* isomers (peak **2**) convert to *ZZ* isomers (peak **3**) at lower collision zone potential differences thanto *EE* isomers (peak **1**). The potential energy surface barriers are also consistent with the fact that collisional excitation of *ZZ* isomers (peak **3**) generated a small amount of *EZ* isomers (peak **2**) at intermediate potential differences, and with a contribution of the *ZE* isomer to ATD peak **1**.

### Photoisomerization of S9M

**Figure 6:**
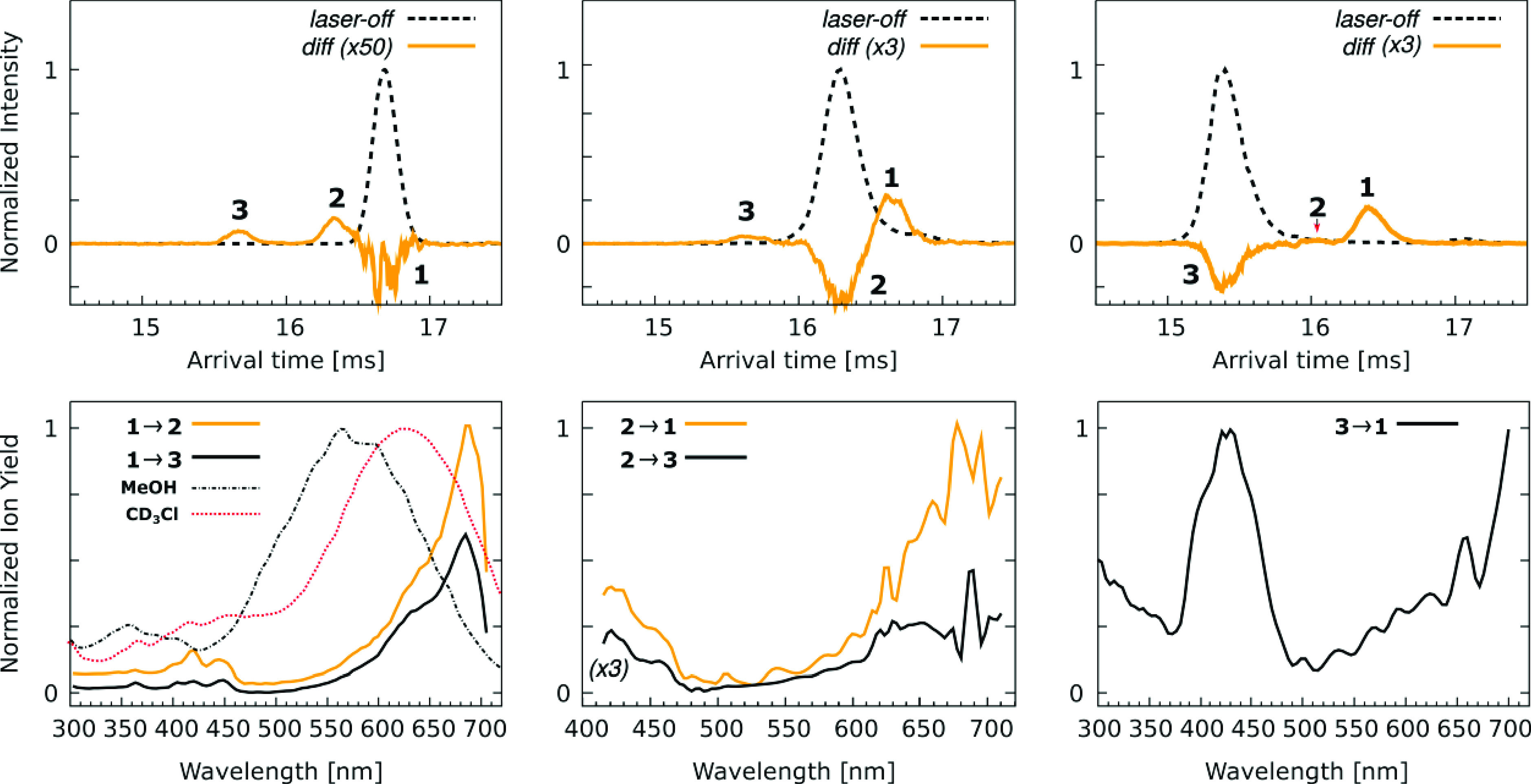
Photoisomerization of selected S9M isomers. (Upper row) Photoaction ATDs for selected ATD peaks **1**, **2**, and **3** (left to right). The excitation wavelengths were 650 nm (peak 1), 430 nm (peak 2), and 420 nm (peak 3). (Lower row) Photoisomerization action (PISA) spectra for selected isomers corresponding to peaks **1**, **2**, and **3** (left to right). In the lower left panel action spectra for isomers associated with peak **1** are compared with absorption spectra for S9M in methanol (black dot-dashed curve) and chloroform (red dotted curve). Note that the photoaction ATDs shown in the upper row were recorded with the laser wavelength tuned to corresponding PISA spectrum maximum.

Photoaction ATDs and photoisomerization action (PISA) spectra for isomers associated with ATD peaks **1**–**3** are shown in Figure 6. The PISA spectra were recorded using the IMS–photo–IMS strategy, which involved separating isomers in the first stage of the drift region, selecting a target ion packet by briefly opening the Bradbury–Nielsen ion gate at an appropriate delay, irradiating the selected isomer packet and separating the photoisomers in the second stage of the drift region. Data were recorded at low light fluences (<1 mJ cm^–2^ for each pulse) such that the photoisomerization yield was no more than a few percent and which should correspond to a single-photon absorption regime. The upper row of plots in Figure 6 shows “light off” ATDs (black dashed curves) and photoaction ATDs (“light on”–“light off”, orange solid curves). The photoaction ATDs show depletion of the parent ATD peak and growth of photoisomer ATD peaks. The lower row of plots shows the PISA spectra associated with photoisomer formation (photoisomer yield plotted against wavelength). Note that due to the limited resolution of each IMS stage it is not possible to individually select peaks **1a** and **1b** isomers or to resolve them as photoproducts. Measurements for ATD peak **1**, which is assumed to be predominantly the *EE* isomer, were performed with low RF voltage applied to IF1 and show formation of isomers corresponding to ATD peaks **2** and **3** (*EZ* and *ZZ*, respectively). Measurements for ATD peaks **2** and **3** were performed with high RF voltage applied to IF1. The photoaction ATD for peak **2** (*EZ*), shows production of photoisomers consistent with ATD peak **1** (*EE*) and peak **3** (*ZZ*), while the photoaction ATD for peak **3** (*ZZ*) shows a photoisomer peak consistent with peak **1** (presumably *ZE*) but no sign of ions associated with peak **2** (*EZ*).

The PISA spectra for ATD peak **1** feature a strong band with a maximum at ∼680 nm and a weaker band peaking at ∼430 nm (Figure 6). Spectra recorded for the **1** → **2** and **1** → **3** photoconversion channels have a similar shape, although the yield of **2** is around twice that of **3**. The **1** → **2** transformation is consistent with *EE* → *EZ* isomerization, while the **1** → **3** transformation is possibly due to *ZE* → *ZZ* isomerization since the *ZE* isomer coexists with *EE* isomers in ATD peak **1**. The **1** → **2** and **1** → **3** PISA spectra are consistent with DF-CC2/cc-pVDZ calculations for the vertical electronic transitions (Table 2), which for *EE*(B) predict bands at 669 and 380 nm with relative intensities of 20:1, respectively. Note that we were unable to record PISA spectra for wavelengths longer than 700 nm due to the limited range of the OPO system. The PISA spectra for ATD peak **1** can be compared to the solution spectra for S9M in methanol and chloroform (Figure 6). In solution, the main absorption band has a maximum at 560 nm (methanol) and 620 nm (chloroform), which are substantially blue-shifted compared to the gas-phase PISA spectra (680 nm).

The PISA spectra for peak **2** (assigned to the *EZ* isomer) recorded for the **2** → **1** (*EZ* → *EE*) and **2** → **3** (*EZ* → *ZZ*) channels are both similar, with the **1** yield being around three times that of **3**. The maximum for the S_1_ ← S_0_ band is at ∼680 nm. The S_2_ ← S_0_ band, present at wavelengths shorter than 470 nm, is more intense than in the PISA spectra for ATD peak **1**. The band wavelengths and intensities in the PISA spectra for ATD peak **2** are consistent with vertical excitation wavelengths and oscillator strengths calculated for the *EZ*(B) and *EZ*(C) conformers (Table 2).

The PISA spectrum for peak **3** recorded on the **3** → **1** channel indicates that the S_1_ ← S_0_ vertical transition occurs at a wavelength longer than 700 nm and that the S_2_ ← S_0_ band has a maximum response at ∼430 nm. These spectral characteristics are consistent with calculated vertical excitation wavelengths and oscillator strengths for the most probable conformers contributing to Peak **3**, *ZZ*(C) and *ZZ*(D) (see Tables 1 and 2).

### Role of Ground-State Isomerization

Following absorption of light, the S9M ions can undergo *E–Z* isomerization about one or both of the linking alkene double bonds by passage through a conical intersection with subsequent collisions relaxing and stabilizing the nascent photoisomer. However, both photoisomers and non-isomerized molecules will possess significant vibrational energy following photoexcitation and nonradiative decay so that it is also possible that rapid statistical isomerization occurs on the ground electronic state potential energy surface before collisional cooling by buffer gas molecules has stabilized the nascent photoisomers. To help ascertain contributions from secondary statistical isomerization, CME modeling was performed starting with a population of a single rotamer assuming a temperature *T* = 300 K plus the energy imparted through absorption of one or two photons at 430 or 680 nm. These wavelengths are close to the band maxima in the PISA spectra shown in Figure 6. Results of the CME calculations are described in detail in the Supporting Information. Significantly, the photoactivated *EE* and *ZE* ions are predicted by the CME modeling to rapidly interconvert before collisional stabilization with the branching ratio depending on the number and energy of the absorbed photons. Given that both *EE* and *ZE* isomers probably contribute to ATD peak **1**, this secondary statistical isomerization process will probably not be apparent in the photoaction ATDs. For the *EZ* and *ZZ* isomers, statistical isomerization to the *EE* or *ZE* forms is predicted to only play a role following absorption of two photons at 680 or 430 nm. We expect that two-photon absorption should be minimal for the data shown in Figure 6, which were recorded for relatively low laser power. In summary, the CME modeling predicts that secondary statistical isomerization plays at most a minor role in the photoisomerization of S9M ions in the gas phase, implying that photoisomerization is driven through passage through conical intersections that link the excited state and ground state potential energy surfaces.

### Photoisomerization Pathways and Comparison with Congo Red

It is interesting to compare the photoisomerization behavior of S9M with that of Congo Red, for which it was observed that absorption of a single visible photon exclusively provoked *E–Z* isomerization about only one of the two equivalent double bonds—single photon *EE* → *ZZ* or *ZZ* → *EE* photoisomerization processes do not occur.^40^ The situation is less clear for S9M, due to the fact that there are four isomers, two of which (*EE* and *ZE*) have similar energies and CCSs, and which are difficult to cleanly separate as targets or as photoproducts. Assuming for the moment that absorption of a single photon only causes isomerization about one of the two double bonds, one could rationalize the photoconversion of peak **1** to peak **3** as arising from *ZE* → *ZZ* photoisomerization. On the other hand, the fact that peak **3** exclusively yields peak **1** would imply that *ZZ* → *ZE* photoisomerization occurs, but that *ZZ* → *EZ* photoisomerization, which would give rise to peak **2**, does not. This would imply that photoisomerization is more facile about the alkene bond connecting the methylbenzothiazolium moiety and the central cyclohexenyl group than about the alkene bond connecting the (dimethylamino)phenyl group with the cyclohexenyl group. However, this would be somewhat odd given the observed photoconversion of peak **2** to peak **3**, which implies that *EZ* → *ZZ* photoisomerization takes place. The alternative is that absorption of a single photon can cause *E–Z* isomerization of both alkene bonds. This might explain the peak **3** to peak **1** photoconversion (*ZZ* → *EE*) and is also consistent with peak **1** to peak **3** photoconversion (*EE* → *ZZ*), but prompts the question of why the former process appears to be 100% efficient while the latter process competes with peak **1** to peak **2** photoconversion (*EE* → *EZ*). Ultimately, to properly tease out the photoconversion pathways, it would be necessary to conduct tandem IMS-photo-IMS studies in which all four geometric isomers are cleanly resolved, preferably also with resolution of the conformers/rotamers associated with each isomer.

## Conclusions

This work demonstrates that isolated S9M can exist in several stable isomeric forms, with the predominant gas-phase isomer having both alkene linkages in their *E* configurations. Using tandem ion mobility spectrometry coupled with laser spectroscopy, we demonstrated photoinduced interconversion between each isomeric form following the absorption of light over the 600–700 nm range. Calculations suggest that the *ZZ* isomer exhibits a π-stacked conformation and an enhanced S_2_ ← S_0_ absorption band compared to the other geometric isomers, consistent with the experimental observations. Subjecting selected S9M isomers to energetic collisions with buffer gas molecules drives a one-way cascade toward the lowest energy *EE* isomer. In contrast, the absorption of light is able to drive reversible isomerization about the two alkene double bonds through processes that involve passage through conical intersections rather than through statistical isomerization on the ground state potential energy surface. Overall, the current study highlights the multipathway character for geometric transformations in a potential styryl-based photoswitch, and demonstrates the use of ion mobility spectrometry coupled with laser spectroscopy to selectively study the conversion between different geometric forms of charged molecules in the gas phase.

There are several avenues for further work. First, a better understanding of the potential energy landscape and photophysical behavior of S9M and similar molecules should be possible through ion mobility studies at lower temperature and at higher resolution allowing all relevant conformers to be separated and probed individually.^60,61^ Second, it would be interesting to compare the PISA spectra of S9M ions, which exhibit rather broad bands, with photodissociation action spectra of cold S9M ions tagged with rare gas atoms, which should reflect their intrinsic absorption spectrum. Parallel infrared studies of cold, mobility-selected S9M ions would also provide structural information on the different isomers and conformers.^62,63^ Last, it would be interesting to explore theoretically the mechanisms behind the photoisomerization and whether isomerization about both double bonds can be triggered by the absorption of a single visible photon.

## Supporting Information Available

Structures and relative energies of additional S9M isomers; analysis of statistical isomerization following photoisomerization (PDF)
