# Supplementary material for: Photoisomerization of Linear and Stacked Isomers of a Charged Styryl Dye: A Tandem Ion Mobility Study
Source: J Am Soc Mass Spectrom. 2021 Nov 17;32(12):2842–51. doi: 10.1021/jasms.1c00264 (PMC8640989; doi:10.1021/jasms.1c00264)
Supplement: Supplementary file 1 — js1c00264_si_001.pdf [file js1c00264_si_001.pdf]

SUPPORTING INFORMATION

Photoisomerization of linear and stacked isomers of a charged styryl dye - a tandem ion mobility study

Eduardo Carrascosa[1,†], James N. Bull[1,2], Emilio Martínez-Núñez[3], Michael S. Scholz[1,‡], Jack T. Buntine[1], and Evan J. Bieske[1,*]

[1]School of Chemistry, The University of Melbourne, Parkville, Victoria 3010, Australia

[2]James N. Bull: School of Chemistry, Norwich Research Park, University of East Anglia, Norwich NR4 7TJ, United Kingdom

[3] Departamento de Química Física, Universidade de Santiago de Compostela, 15782 Santiago de Compostela, Spain

† Present address : Laboratoire de Chimie Physique Moléculaire, École Polytechnique Fédérale de Lausanne, EPFL SB ISIC LCPM, Station 6, CH-1015 Lausanne, Switzerland

* Evan J. Bieske; e-mail: evanjb@unimelb.edu.au

Structures and relative energies of additional Styryl 9M isomers

In addition to the single-bond rotation structures for S9M shown in Figure 2 in the paper, the concerted rotation of both single bonds generates further conformers, labelled as the D structures (Figure S1). Except for the *ZZ*(D) structure, which is predicted to be the lowest energy *ZZ* conformer, the *EE*(D), *ZE*(D) and *EZ*(D) conformers are substantially less stable than their corresponding (A), (B) or (C) structures. Potential three-ring cyclic structures resulting from stilbene-type cyclization were considered during the computational evaluation. The cyclic forms have two families, defined by whether the adjacent hydrogens on the cyclized ring point in the same or opposite directions. The lowest energy cyclic conformer, which lies 0.68 eV above the *EE*(B) isomer, is shown in Figure S1. Ultimately, these cyclic isomers were deemed to be unimportant.

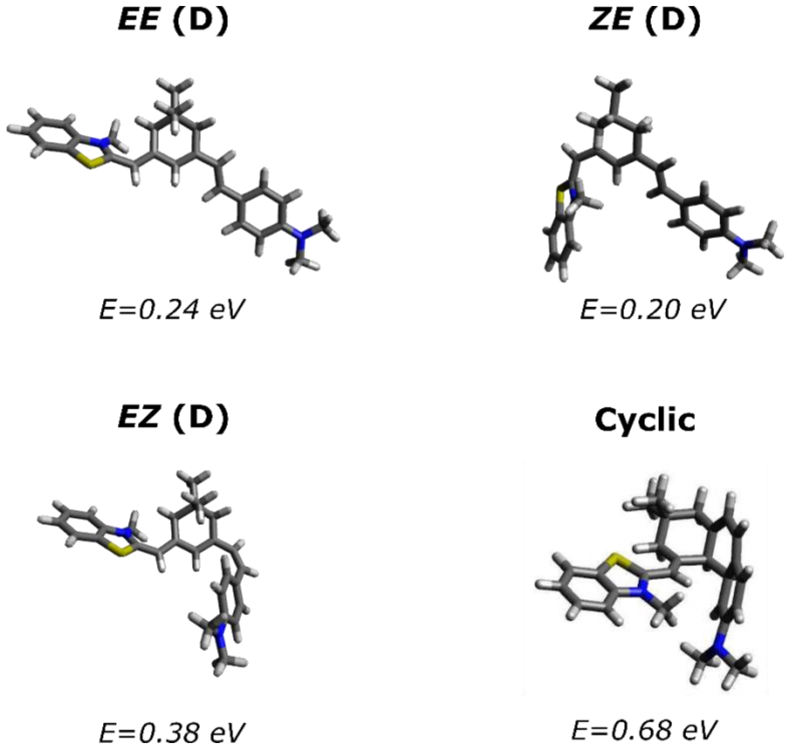

Figure S1: Structures and relative energies [with respect to *EE*(B)] of three high energy conformers of the *EE*, *ZE*, and *EZ* structures, and the lowest energy cyclic structure. Relative energies were computed at the DLPNO-CCSD(T)/cc-pVTZ//ωB97X-D/cc-pVDZ level of theory.

Statistical Isomerization after Photoisomerization

Following excitation of either the $S_1 \leftarrow S_0$ or $S_2 \leftarrow S_0$ transition, rapid isomerization by passage through a conical intersection connecting the ground and excited potential energy surfaces is likely to be the primary isomerization mechanism. However, both photoisomers and non-isomerized molecules are expected to have significant vibrational energy following photoexcitation and non-radiative decay and may undergo statistical isomerization on the ground state potential energy surface before their internal energy is quenched through collisions. In this section we evaluate the role of such secondary isomerization using statistical reaction rate theory calculations (Chemical Master Equation, CME, approach) implemented within the MESMER 6.0 package.

The results of the CME simulations are shown in Figures S2-S5, which illustrate the evolution of the main S9M rotamers and isomers with different amounts of initial vibrational energy. Simulations were performed assuming the ions have average thermal energy associated with a temperature of T=300K plus the energy imparted through

absorption of one or two photons at 680 nm or 430nm, where these wavelengths correspond to maxima in the PISA spectra. The simulations assumed conformer electronic energies and transition state energies calculated at the DLPNO-CCSD(T)/cc-pVTZ//wB97X-D/cc-pVDZ level of theory (see Figure 5 in the paper).

The simulations show several general features:

(1) At very short times (<ns) a rapid "microcanonical" equilibrium is stablished between the single bond rotamers.
(2) Collisions with the buffer gas, occurring at a rate ~2×10^{8} s^{-1}, begin to remove vibrational energy from the ion.
(3) While the total internal energy decays exponentially, trending towards an asymptote corresponding to the thermal energy, there is a range of times over which the ions have sufficient energy to traverse barriers separating different isomers (tens to hundreds of nanoseconds). By ~5×10^{-6} s, the ions have undergone several hundred collisions and are close to being thermalized.
(4) At high internal energies (e.g. following absorption of two photons), statistical isomerization over *E*-*Z* barriers (transition states) is more rapid than collisional quenching, leading to formation of several isomers. For example, *EE* and *ZE* isomers are formed irrespective of the starting isomer.

Individual transformations are discussed in more detail below.

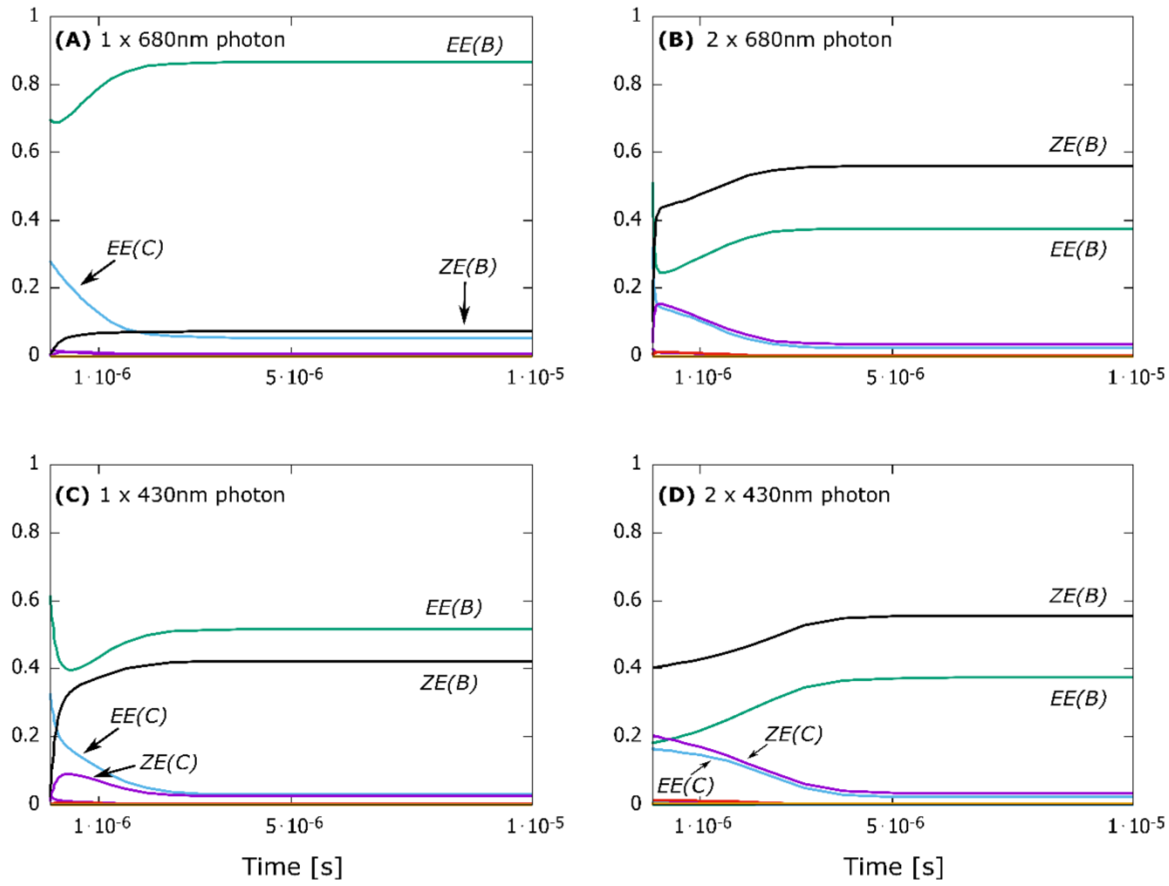

Figure S2: CME simulations of isomer abundances with time with an initial population of *EE*(B) at internal energies equivalent to (A) *T* = 300 K + one 680 nm photon; (B) *T* = 300 K + two 680 nm photons; (C) *T* = 300 K + one 430 nm photon; (D) *T* = 300 K + two 430 nm photons.

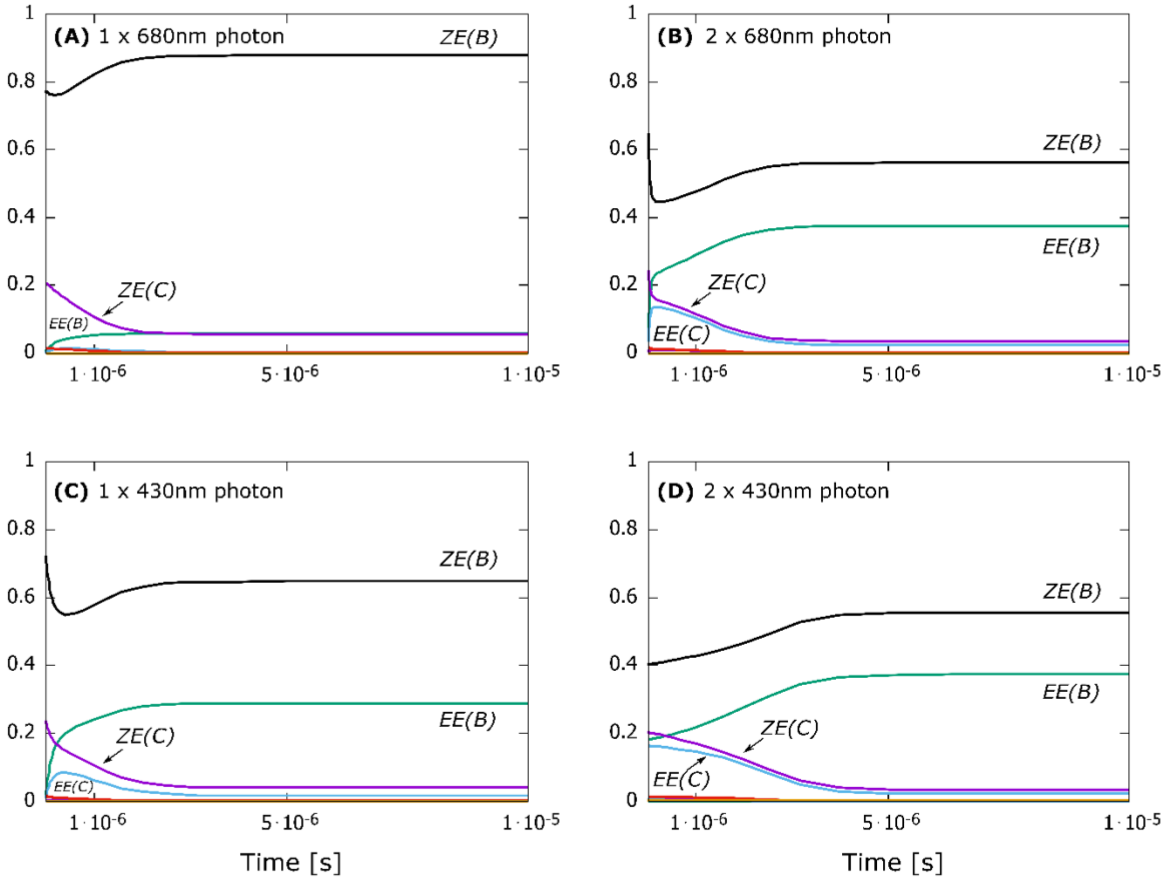

Figure S3: CME simulations of isomer abundances with time with an initial population of *ZE*(B) at internal energies equivalent to (A) *T* = 300 K + one 680 nm photon; (B) *T* = 300 K + two 680 nm photons; (C) *T* = 300 K + one 430 nm photon; (D) *T* = 300 K + two 430 nm photons.

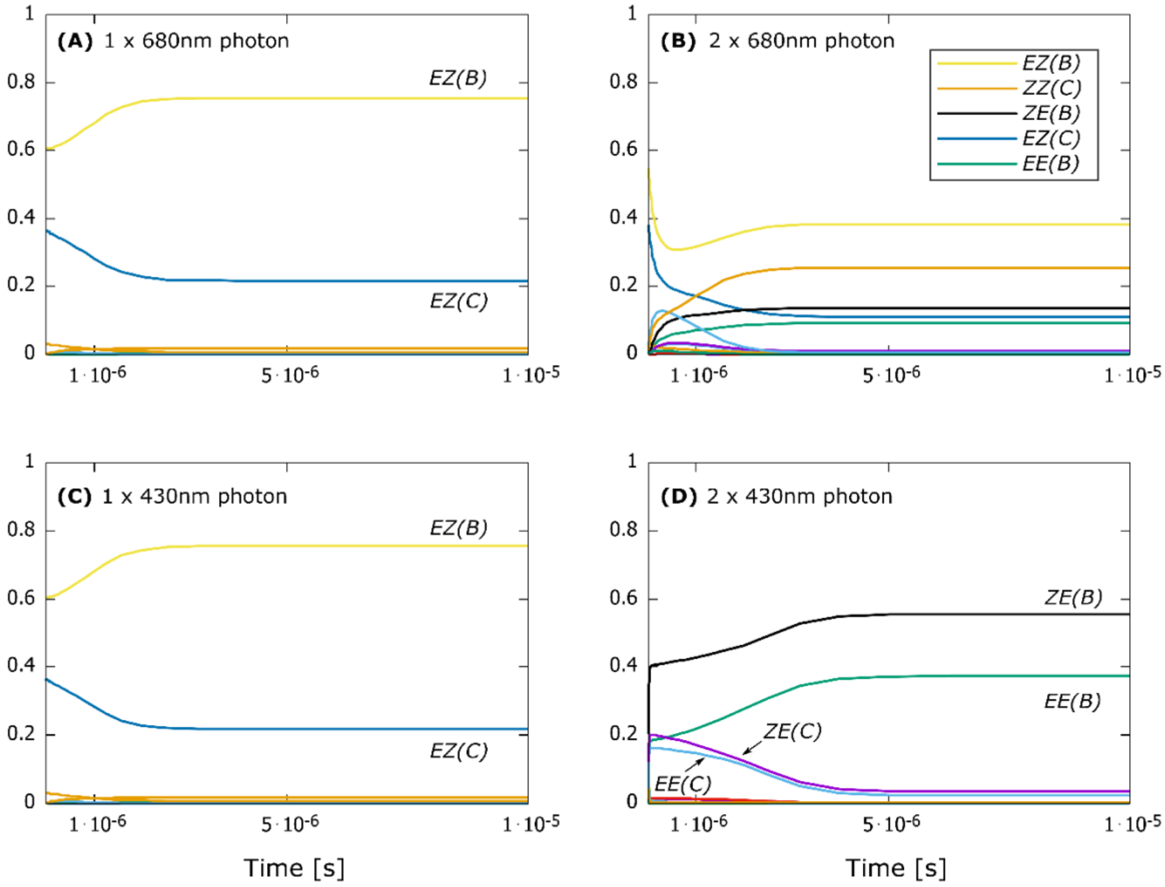

Figure S4: CME simulations of isomer abundances with time with an initial population of *EZ*(C) at internal energies equivalent to (A) T = 300 K + one 680 nm photon; (B) T = 300 K + two 680 nm photons; (C) T = 300 K + one 430 nm photon; (D) T = 300 K + two 430 nm photons.

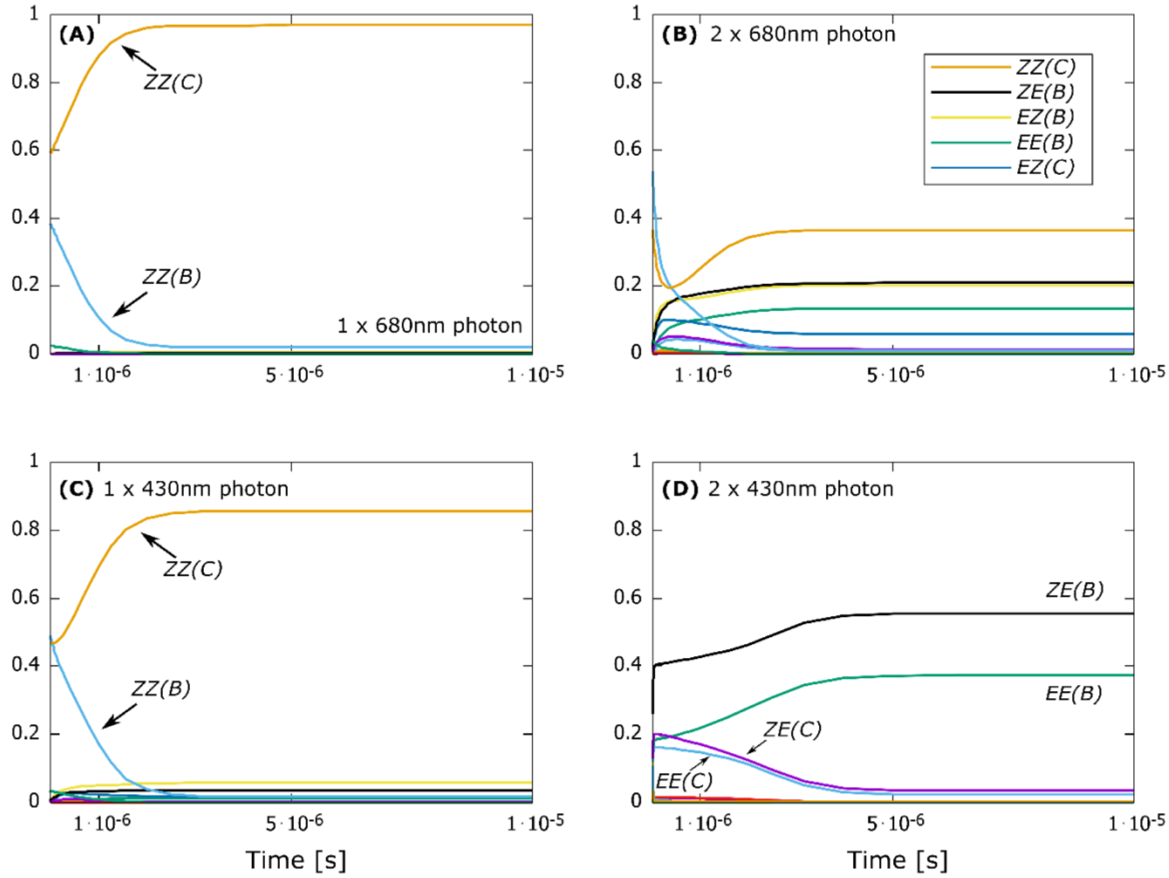

Figure S5: CME simulations of isomer abundances with time with an initial population of *ZZ*(C) at internal energies equivalent to (A) T = 300 K + one 680 nm photon; (B) T = 300 K + two 680 nm photons; (C) T = 300 K + one 430 nm photon; (D) T = 300 K + two 430 nm photons.

For nascent *EE*(B) isomers activated with one or two photons (Figure S2), rapid statistical interconversion between *EE*(B) and *ZE*(B) isomers occurs, with the *ZE*(B) yield higher than the *EE*(B) yield under two photon absorption conditions. The preference for *ZE*(B) with two photon absorption is because of a higher density of states for this isomer, which influences the equilibrium constant between *ZE(B)* and *EE(B)* more than the difference in ZPE-corrected energy between both species. An exception exists for the initial internal energy corresponding to absorption of a single 680nm photon (Figure S2(A)), where a minor fraction of *EE*(C) is present because it is initially favoured during the microcanonical equilibrium and survives collisional quenching on the microsecond timescale.

For nascent *ZE*(B) isomers activated with one or two photons (Figure S3), the situation is similar to that for *EE*(B), with a small degree of interconversion between *ZE*(B) and *EE*(B) isomers at lower internal energies, and reaching the same isomer branching ratios for two-photon absorption. Again, a minor fraction of the population (<10%) remains as the *ZE*(C) isomer for internal energies corresponding to absorption of a single 680nm photon (Figure S3(A)). The *ZE*(C) isomer is formed during the initial rapid equilibration on the picosecond timescale.

In the case of the *EZ*(C) isomer (Figure S4), an initial equilibration with the energetically more stable *EZ*(B) isomer is established after absorbing a single 680 nm or 430 nm photon, leading to a population of >75% of *EZ*(B) ions after tens of microseconds. Ultimately, because the internal energy changes with wavelength, the degree of statistical isomerization will differ across the PISA spectra.

ZZ(C) isomers activated by a single 680 nm photon undergo collisional quenching before statistically isomerizing to other geometries (Figure S5(A)). In contrast, *ZZ*(C) isomers activated by a single 430 nm photon statistically isomerize to form small quantities of the *EZ*(B) and *ZE*(B) isomers; this isomerization process is assisted by an initial *ZZ*(C) $\rightarrow$*ZZ*(B) single bond rotation. Similar to the *EZ*(C) isomer, the *ZZ*(C) isomer activated by two 680 nm photons is predicted to produce <40% *ZZ*(C), ≈20% both *ZE*(B) and *EZ*(B), and ≈10% *EE*(B). As explained above, the experimental conditions make multiphoton (sequential) absorption at these wavelengths unlikely. Simulations

assuming absorption of two 430 nm photons show production of only *ZE*(B) and *EE*(B) isomers. Under these conditions photoaction ATD peak **1** would be enhanced compared to peak **2**. Note that the *ZZ*(D) isomer was not included in these simulations because the simulations were intended to evaluate whether the PISA results are possibly affected by statistical isomerization, and given that *ZZ*(C) and *ZZ*(D) are associated with the same ATD peak **3**.
